# A novel bioreactor system for biaxial mechanical loading enhances the properties of tissue-engineered human cartilage

**DOI:** 10.1038/s41598-017-16523-x

**Published:** 2017-12-05

**Authors:** Christoph Meinert, Karsten Schrobback, Dietmar W. Hutmacher, Travis J. Klein

**Affiliations:** 1 Institute of Health and Biomedical Innovation, Queensland University of Technology, Brisbane, Queensland 4059 Australia; 2 Australian Research Council Industrial Transformation Training Centre in Additive Biomanufacturing, Queensland University of Technology, Brisbane, Queensland 4059 Australia

## Abstract

The *ex vivo* engineering of autologous cartilage tissues has the potential to revolutionize the clinical management of joint disorders. Yet, high manufacturing costs and variable outcomes associated with tissue-engineered implants are still limiting their application. To improve clinical outcomes and facilitate a wider use of engineered tissues, automated bioreactor systems capable of enhancing and monitoring neotissues are required. Here, we developed an innovative system capable of applying precise uni- or biaxial mechanical stimulation to developing cartilage neotissues in a tightly controlled and automated fashion. The bioreactor allows for simple control over the loading parameters with a user-friendly graphical interface and is equipped with a load cell for monitoring tissue maturation. Applying our bioreactor, we demonstrate that human articular chondrocytes encapsulated in hydrogels composed of gelatin methacryloyl (GelMA) and hyaluronic acid methacrylate (HAMA) respond to uni- and biaxial mechanical stimulation by upregulation of hyaline cartilage-specific marker genes. We further demonstrate that intermittent biaxial mechanostimulation enhances accumulation of hyaline cartilage-specific extracellular matrix. Our study underlines the stimulatory effects of mechanical loading on the biosynthetic activity of human chondrocytes in engineered constructs and the need for easy-to-use, automated bioreactor systems in cartilage tissue engineering.

## Introduction

Tissue engineering is a promising alternative to present surgical cartilage repair techniques which often fail to restore long-term physiological tissue function^1,2^. Current research efforts aim to generate biological substitutes that recapitulate the morphological, biochemical, as well as mechanical properties of native articular cartilage^3^ using a combination of cells and biomaterials, as well as external stimulation through biological and/or mechanical factors^4^. Chondrocytes and other cells used to engineer cartilage respond to mechanical loading via mechanotransduction, a process by which physical stimuli are sensed and converted to biochemical signals that regulate fundamental cellular behaviours. It is generally accepted that mechanical loading at physiologically low levels stimulates the biosynthesis of extracellular matrix (ECM) macromolecules^5^ to improve the mechanical and biochemical properties of engineered cartilage neotissues^6,7^. This has motivated the design of various bioreactor systems^8^ aiming to provide mechanical stimuli which favour tissue maturation under tightly controlled and monitored loading conditions. The working principles of such systems involve, for example, the application of hydrostatic pressure^9,10^, compressive^11,12^ or shear loading^13,14^. Yet, only a small number of publications report on the application of biaxial loading bioreactors enabling reproducible and well-controlled changes to specific loading parameters^15–17^, none of which are commercially available.

Despite significant progress in the field^18^, the *ex vivo* generation and clinical use of cartilage tissues is still impeded by a limited understanding of the influence of specific physicochemical culture parameters on tissue development, as well as the high manufacturing costs associated with autologous tissue-engineered products^19^. By providing a high level of process control and automation, bioreactor systems have the potential to both, identify optimal culture conditions promoting the maturation of cartilage neotissues, and significantly reduce the associated costs^19^. Furthermore, the role of mechanical stimulation bioreactors can be broadened beyond the conventional approach of enhancing the quality of tissue-engineered cartilage constructs^19^. For example, they can also serve as valuable *in vitro* models to study the pathophysiological effects of physical forces involved in the onset of osteoarthritis, or to study basic phenomena such as mechanotransduction. Yet, the design of such a system has to be carefully considered. In order to study the basic mechanobiology of chondrocytes in a physiologically relevant manner, bioreactors should be able to apply compression and shear - the main types of loading articular cartilage experiences during day-to-day usage^20^. In addition, bioreactors should ideally also consider more practical aspects of tissue culture and allow for easy assembly, as well as cleaning and sterilization. Systems must accommodate a relevant number of samples to allow for sufficient statistical power in the data analysis and provide a high level of automation with a user-friendly and robust interface. Another important consideration is the capability to monitor the functional maturation of cartilage neotissues through non-destructive mechanical testing. Devices implementing these and other design features have the potential to automate cartilage tissue manufacture under standardized and controlled physicochemical environmental conditions, in turn reducing production costs and facilitating a wider use of *ex vivo* engineered tissues for clinical cartilage repair.

The success of *ex vivo* cartilage tissue engineering applications is similarly dependent on the capacity of biomaterials to promote chondrogenic differentiation and cartilage ECM synthesis. Hydrogels derived from native ECMs are increasingly applied for these purposes as they inherently retain cell-attachment motifs, are enzymatically degradable, and possess cell-instructive bioactivity^21^. For example, gelatin methacryloyl (GelMA) hydrogels have attracted great attention in recent years^22^ due to their excellent bioactivity, biocompatibility, as well as their non-immunogenic, tuneable properties, and ease of manufacture^23^. GelMA is produced by the chemical functionalization of gelatin and can be covalently crosslinked under gentle conditions, allowing the encapsulation of chondrocytes with high viability^24^. By copolymerization of small quantities of methacrylate-functionalized hyaluronic acid (hyaluronic acid methacrylate, or HAMA), chondrogenic re-differentiation of expanded human chondrocytes can be significantly enhanced, leading to hyaline cartilage-like neotissue formation with steadily increasing mechanical strength^24^. Despite these promising results, it remains unclear how encapsulated chondrocytes react to physiological mechanical stimuli in this hydrogel system - an important factor which warrants further investigation for potential clinical translation.

In this study, we have implemented the aforementioned design considerations to develop an innovative bioreactor system capable of applying precise uni- or biaxial mechanical stimulation to developing cartilage neotissues in a tightly controlled and monitored environment. Employing this system, we investigated the effects of pre-culture and various uni- and biaxial loading regimes on human chondrocyte gene expression in GelMA-HAMA hydrogels. We have further performed long-term experiments to investigate the effects of biaxial mechanical loading on cartilage neotissue formation. Our results demonstrate that uniaxial shear and compression, as well as biaxial stimulation promote the expression of chondrogenic marker genes. We further demonstrate that intermittent biaxial stimulation facilitated by our system significantly enhances the accumulation of cartilage-specific ECM.

## Results

### Shear and compression bioreactor system

**Figure 1:**
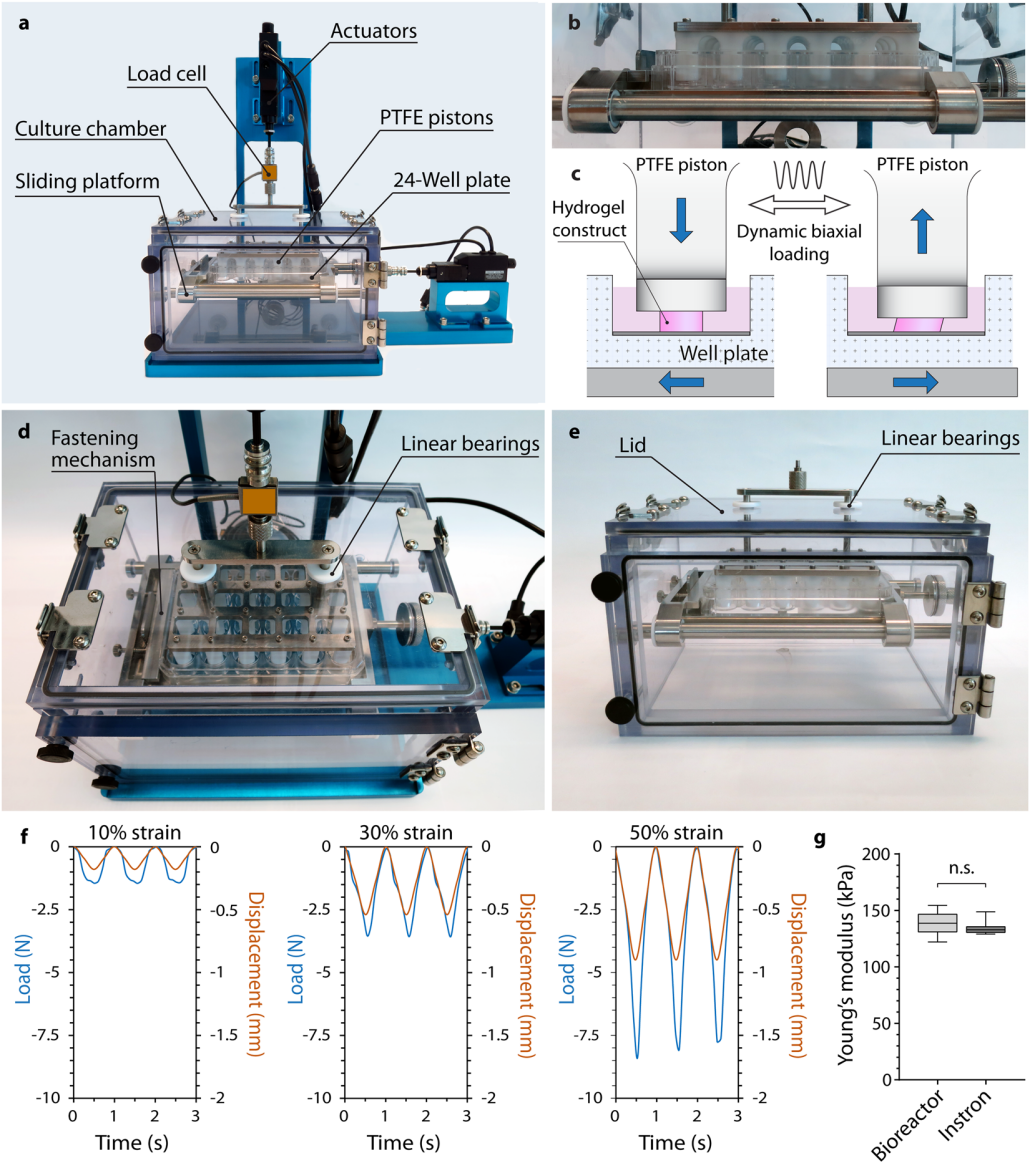
Mechanical stimulation bioreactor system. (**a**) Front view depicting the bioreactor stand (anodized in blue) and the removable polycarbonate culture chamber. (**b**) Close-up view of PTFE pistons lowered into a standard 24-well plate on the actuator-driven sliding platform. (**c**) Compressive loading is facilitated by actuator-driven PTFE pistons, while the movement of the sliding platform results in shear deformation of hydrogel construct fully immersed in culture media. (**d**) Top-view of the culture chamber with PTFE bearings and the spring-loaded fastening mechanism used to secure cell culture plates. (**e**) Culture chamber with front door and removable lid. (**f**) Representative force-displacement curves recorded with the bioreactor’s 50 N load cell for 10%, 30%, and 50% dynamic compressive strain applied to 6 hydrogel samples (1 Hz) placed in a 24-well plate. (**g**) Comparison of Young’s moduli of 15% GelMA hydrogels determined using the bioreactor system or an Instron Microtester demonstrating the systems’ capability for mechanical testing (n = 7, p = 0.443).

The bioreactor was designed to apply uni- or biaxial sliding shear and compressive stimulation to a variety of sample types such as cartilage explants or cell-hydrogel constructs for tissue engineering (Supplementary Video V1), but can be readily adapted to various other applications or sample types. The device is subdivided into two main structural components, (i) the stand and base plate, and (ii) the polycarbonate culture chamber (Fig. 1a). The aluminium stand and base plate were anodized to prevent corrosion and carry two orthogonally aligned micro linear actuators with a resolution (microstep size) of <50 nm and a repeatability of <1 μm to facilitate biaxial mechanical stimulation, translating to a unidirectional accuracy of 15 μm. The actuators allow for a travel range of 25 mm, up to 8 mm/s speed and 50 N continuous thrust, and provide real-time position feedback by integrated controllers. Static or dynamic compression is facilitated by PTFE plungers attached to a vertically driven loading plate (Fig. 1b,c, Supplementary Video V1). A modular design allows for rapid exchange of the loading plate and plungers, permitting the use of various standard cell culture plates, such as 24- or 48-well plates. The culture plates are placed on a sliding platform which is driven horizontally on stainless steel rods with self-lubricating PTFE linear bearings to enable the exertion of precise shear stimulation while minimizing effects of friction and inertia (Fig. 1b, Supplementary Video V1). To ensure backlash-free movement, the well plate is secured by a spring-loaded fastening mechanism (Fig. 1d). All mechanical feed-throughs are equipped with PTFE bearings to minimize friction during actuator movement. Parallel and angular misalignments of the actuators relative to the loading plate or sliding platform are corrected by specially developed components comprising two stainless steel parts with a tangential globular and a flat surface, respectively, surrounded by a firm piece of tubing. The bioreactor chamber and interior were manufactured from autoclavable materials (PTFE, stainless steel, and polycarbonate) and allow for easy cleaning to ensure aseptic long-term tissue culture. Custom designed connectors facilitate quick detachment of the chamber from the actuators without the necessity of tools to enable transfer to a biological safety cabinet for sampling or media change. The device is also fitted with a 50_N miniature load cell for providing real-time force feedback during loading and reliable mechanical testing (Fig. 1f,g). Although not further investigated in this paper, the chamber can optionally be equipped with an oxygen sensor and a sterile gas inlet to supply an oxygen-free gas mixture with 5% CO_2_, regulated by a commercial oxygen controller (ProOx P110, Biospherix, NY, USA), enabling experiments under controlled oxygen tension. All gas inlets are protected with gas-permeable 0.2 µm sterile filter units to ensure adequate and aseptic gas exchange. Experiments involving mechanical loading under hypoxic atmosphere require unloaded control samples. Therefore, the bioreactor chamber was designed to accommodate additional well plates underneath the sliding platform which can be accessed through an O-ring sealed door in the front. The assembled device is light (approximately 2.5 kg) and compact (L × W × H: 40 cm × 23 cm × 33 cm), and can be placed in a standard cell culture incubator for long-term culture in a pH- and temperature-controlled environment.

### Actuator control and data acquisition

**Figure 2:**
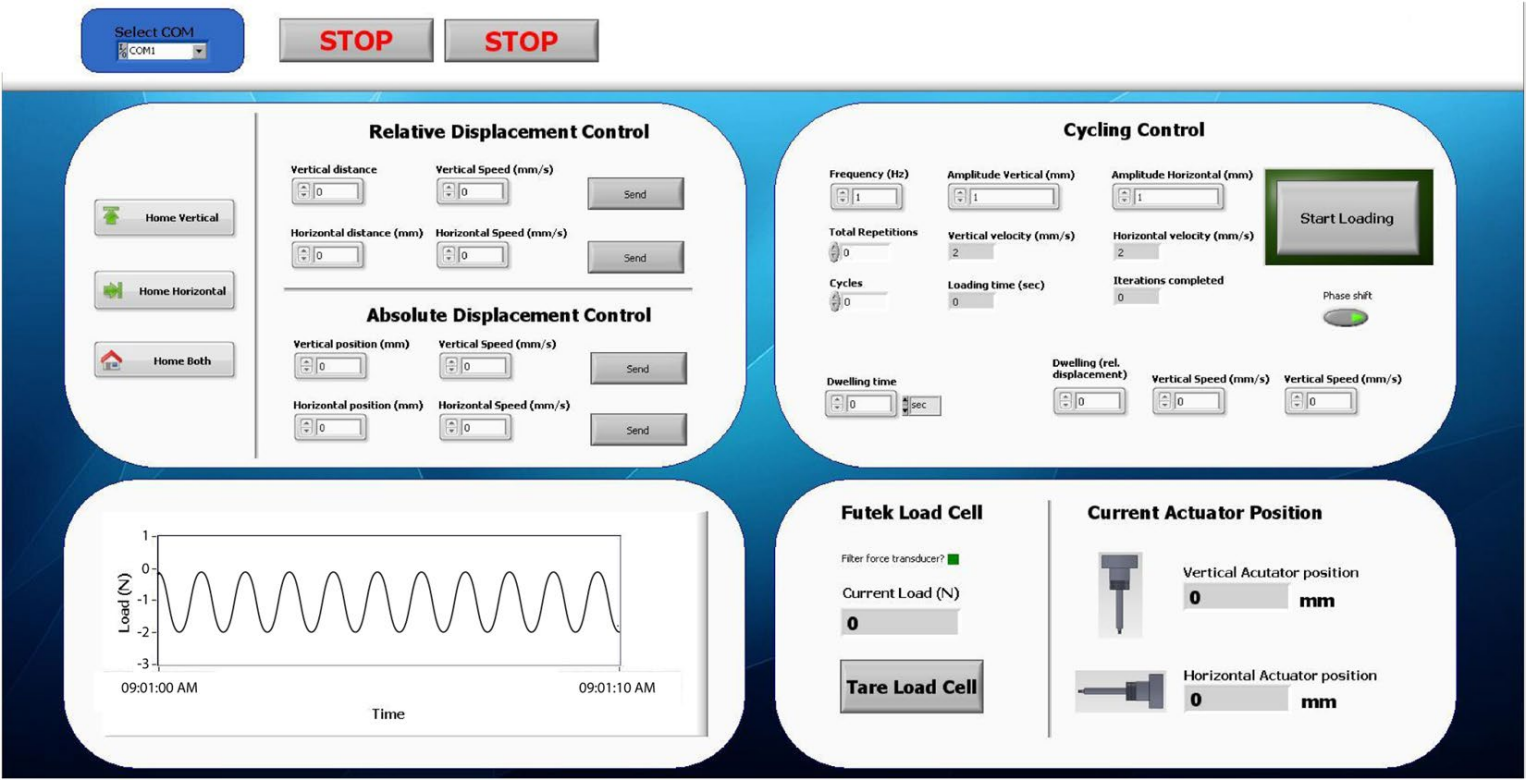
Screenshot of the software graphical user interface used to control the bioreactor system. The custom-developed LabView virtual instrument allows control of actuator position using relative and absolute displacement commands, as well as dynamic uni- or biaxial mechanical stimulation parameters, and provides a graphical interface for force and actuator position feedback.

We developed a user-friendly LabView virtual instrument permitting control of actuator and mechanical stimulation parameters, as well as graphical display and acquisition of load cell and actuator position-readings in real-time (Fig. 2). The software allows for individual absolute actuator target position or relative displacement commands with integrated motion velocity control (from 0.00022 mm/s to 8 mm/s) and permits control of dynamic uni- or biaxial stimulation parameters such as loading frequency, individual amplitudes of shear and compressive loading, waveform (triangular, sinusoidal, square, or ramp), and loading duration. To minimize hands-on time for long-term experiments, it further allows control of total loading cycle repetitions and specification of dwelling time between loading cycles, enabling the bioreactor to automatically apply, for example, 1 hour of dynamic stimulation every day for a specified amount of days. Static compression significantly inhibits chondrocyte biosynthesis^25–27^. We have therefore incorporated parameters to control the actuator position between loading cycles, permitting plungers to be lifted off the samples during idle stages before returning to their initial position and the start of a new loading cycle.

### Effects of pre-culture and loading on chondrocyte gene expression

**Figure 3:**
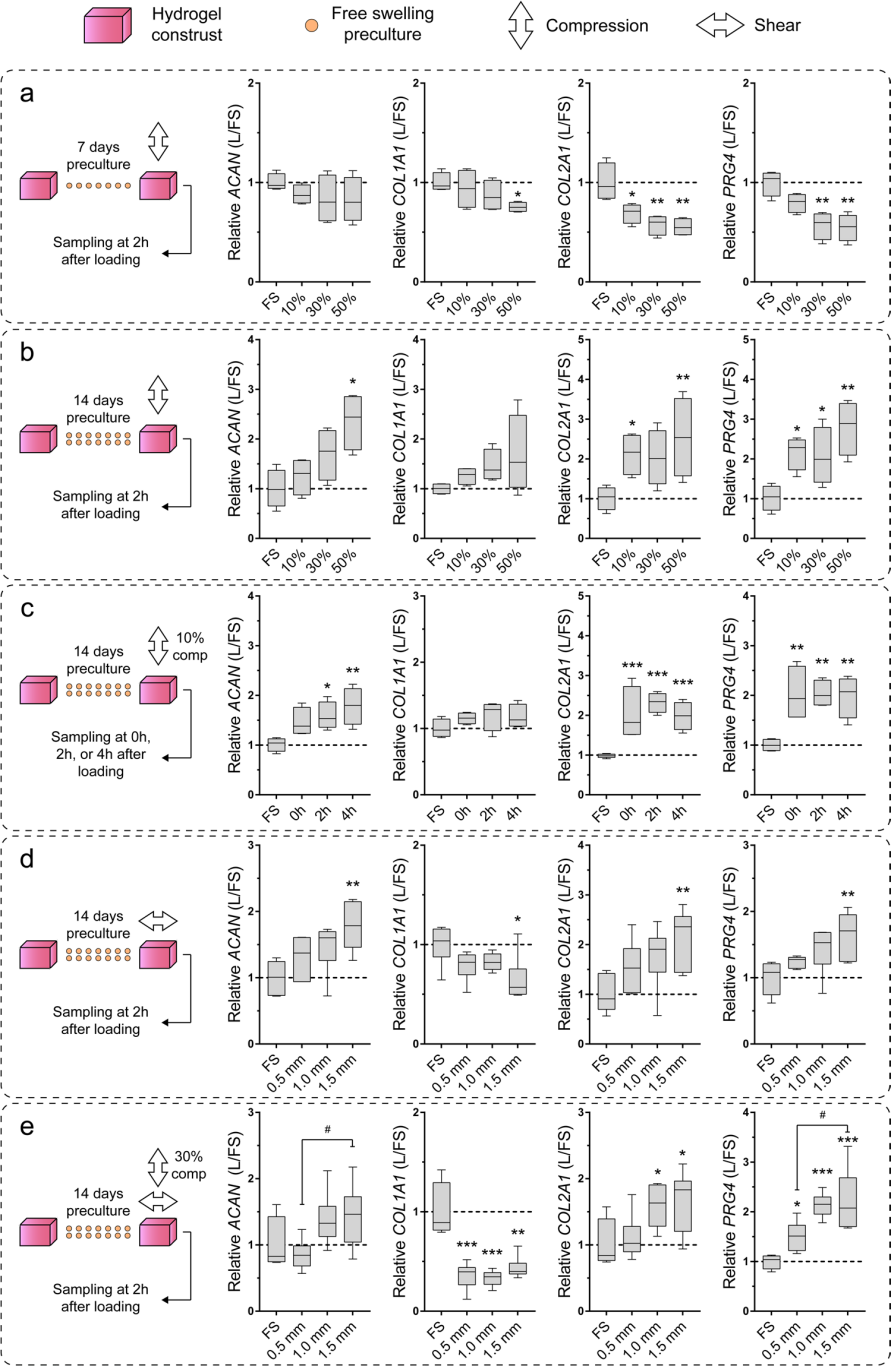
Gene expression following short-term uni- and biaxial mechanical stimulation. Relative expression of chondrogenic (ACAN, COL2A1), de-differentiation (COL1A1), and superficial chondrocyte (PRG4) marker genes following dynamic (**a**–**d**) uni- or (**e**) biaxial stimulation for 1 hour at 1 Hz after (**a**) 7 or (**c**–**e**) 14 days of free swelling preculture. (a + b) Cell-laden GelMA-HAMA constructs were compressed at 10%, 30%, or 50% strain. (**c**) To investigate temporal changes in gene expression, constructs were compressed at 10% and samples taken for total RNA isolation 0 h, 2 h, or 4 h after compression. (**d**) Constructs were stimulated at 0.5 mm, 1.0 mm, or 1.5 mm sliding shear amplitude. (**e**) Constructs were stimulated with 30% compressive strain and either 0.5 mm, 1.0 mm, or 1.5 mm sliding shear amplitude. In all panels, gene expression levels of loaded constructs (L) were normalized to free-swelling controls (FS) cultured for the same duration (mean of free swelling controls indicated by broken line). Stars indicate a statistical difference between loaded and free swelling constructs (*p < 0.05, **p < 0.01, ***p < 0.001), while hashtags indicate statistical differences between loading regimes (#*p* < 0.05) (n = 4–6, one donor per study).

In order to determine suitable culture conditions for cartilage tissue engineering applications, we investigated the effects of pre-culture duration and various uni- and biaxial loading regimes on chondrocyte gene expression. Consistent with our previously published results using alginate hydrogels^28^, compressive loading had no significant (*ACAN*) or only adverse effects on the expression of chondrogenic marker genes *COL2A1* and *PRG4* when cell-laden constructs were pre-cultured for only 7 days before loading (Fig. 3a). When static pre-culture was extended to 14 days, however, there was a trend towards increased transcription of chondrogenic markers *ACAN*, *COL2A1*, and *PRG4*, which was statistically significant at various strain levels compared to free swelling controls (Fig. 3b). To investigate temporal changes of gene expression following compression at 10% strain, constructs were sampled for total RNA isolation either directly after (0 h), or 2 h and 4 h after loading had ceased. Chondrogenic marker genes *COL2A1* and *PRG4* were up-regulated immediately after loading and levels remained elevated for at least 4 h (Fig. 3c). For *ACAN*, significant changes were only observed after 2 h and 4 h, while the expression of *COL1A1* remained at control level at all investigated time points (Fig. 3c). We next investigated the effects of uniaxial shear loading on chondrocyte gene expression. There was a general trend towards up-regulation of chondrogenic markers with increasing shear amplitudes which was significant at 1.5 mm compared to free swelling controls, while the expression of *COL1A1* was down-regulated at this amplitude (Fig. 3d). Dynamic biaxial loading with a compressive strain of 30% and shear amplitudes ranging from 0.5–1.5 mm led to increased transcript levels of all investigated chondrogenic markers *ACAN*, *COL2A1*, and *PRG4*, in an amplitude-dependent manner (Fig. 3e). Interestingly, *COL1A1* expression was significantly lower compared to controls at all investigated shear levels (Fig. 3e). Some signs of wear-and-tear (small cracks and increased surface roughness) were observed on a number of cell-hydrogel constructs which were loaded at 30% compression and 1.5 mm shear amplitude, indicating that this loading regime may be too harsh for tissue engineering applications.

### Effects of intermittent biaxial loading on tissue-engineered cartilage constructs in long-term cultures

#### Cell viability and proliferation in tissue-engineered cartilage constructs

**Figure 4:**
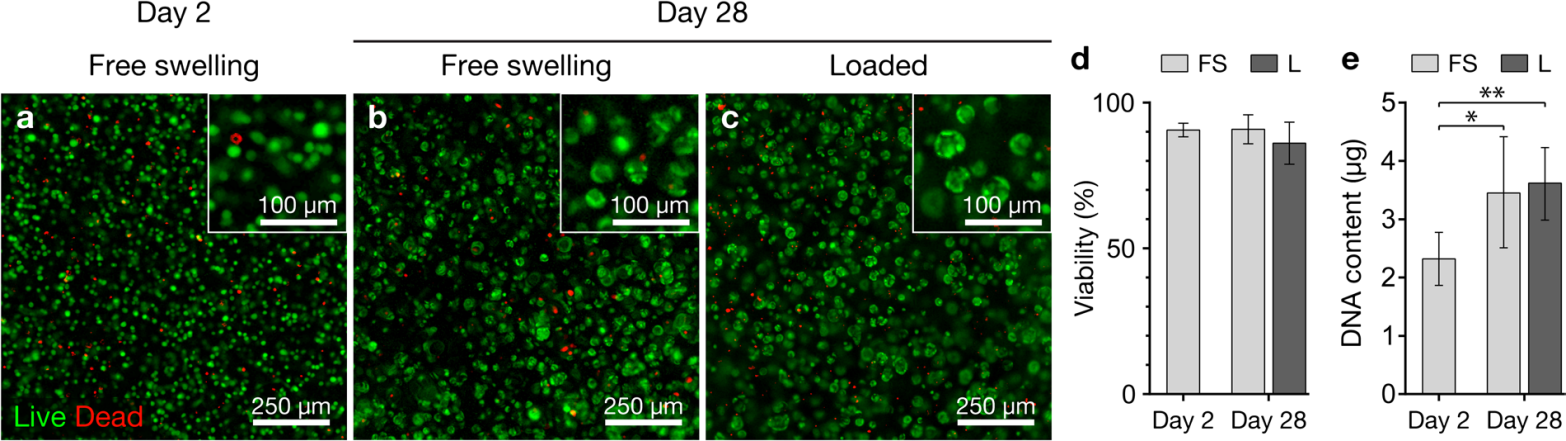
Chondrocyte viability and DNA content of GelMA-HAMA constructs. Viability of chondrocytes at (**a**) day 2 and (**b**) day 28 of free swelling culture, or (**c**) after 14 days of free swelling preculture and 14 days of intermittent biaxial mechanical stimulation at 30% compressive strain and 1 mm shear amplitude (1 Hz, 1 h daily). Living cells appear green, while dead cells appear red. (**d**) Quantified viability (3 donors, 1 construct each) and (**e**) total DNA content (3 donors, 2 constructs each) of free swelling (FS) and loaded (L) constructs. Stars indicate a statistical difference between constructs at day 2 and day 28 of culture (*p < 0.05, **p < 0.01).

We performed long term experiments to investigate whether our bioreactor system can be used to improve the physicochemical properties of tissue-engineered cartilage constructs. Based on the results obtained in the short-term gene expression studies (Fig. 3), cartilage constructs were statically pre-cultured for 14 days, followed by 14 days with intermittent biaxial mechanical stimulation for 1 h daily at 30% compressive strain and 1 mm shear amplitude. Similar to our previously published results using the GelMA-HAMA system without mechanical loading^24^, viability of human chondrocytes was high at all time points (~90%) and no adverse effect of loading was observed (Fig. 4a–d). Chondrocytes appeared as single rounded cells or in small clusters which were evenly distributed throughout the constructs (Fig. 4a–c). The total DNA content increased approximately 1.5-fold in both free swelling and loaded constructs (Fig. 4e).

#### Physical and biochemical properties of tissue-engineered cartilage constructs

**Figure 5:**
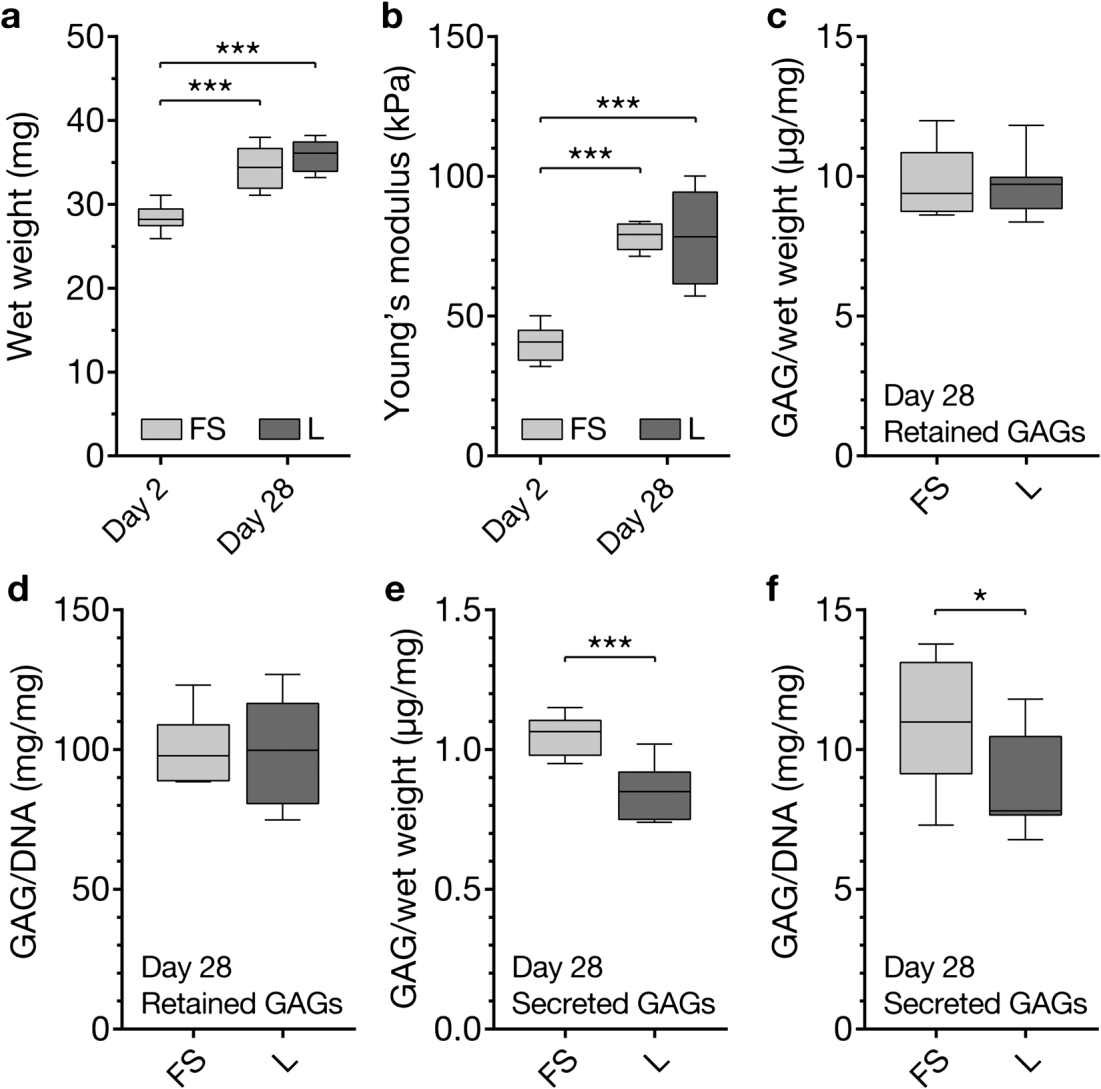
Physical and biochemical properties of tissue-engineered cartilage constructs. Hydrogel construct (**a**) wet weights and (**b**) Young’s moduli at day 2 and day 28 of free swelling culture (FS), or after 14 days of free swelling pre-culture followed by 14 days of intermittent biaxial mechanical stimulation at 30% compressive strain and 1 mm sliding shear amplitude (L) (1 Hz, 1 h daily). Total GAG content retained in the constructs (**c**) normalized to construct wet weight or (**d**) DNA content. GAGs secreted into the media in 24 h with (L) or without (FS) 1 hours of mechanical loading (**e**) normalized to construct wet weight and (**f**) DNA content. In (**a**) and (**b**), stars indicate a statistical difference between constructs at day 28 and day 2, while stars in (**c**–**f**) indicate differences between free swelling and loaded constructs (*p < 0.05, ***p < 0.001) (3 donors, 2 constructs each).

The wet weight of neocartilage constructs increased similarly in both free swelling and loaded cultures (Fig. 5a). Consistent with our previous studies under static culture conditions^29^, the Young’s moduli of neocartilage constructs increased from ~40 kPa to ~80 kPa over 28 days in both free swelling and loaded cultures (Fig. 5b). The accumulation of GAGs in the constructs was not affected by loading (Fig. 5c,d). Interestingly, secretion of GAGs into the culture media was significantly higher in free swelling constructs compared to loaded constructs (Fig. 5e,f).

#### Extracellular matrix protein expression and accumulation

**Figure 6:**
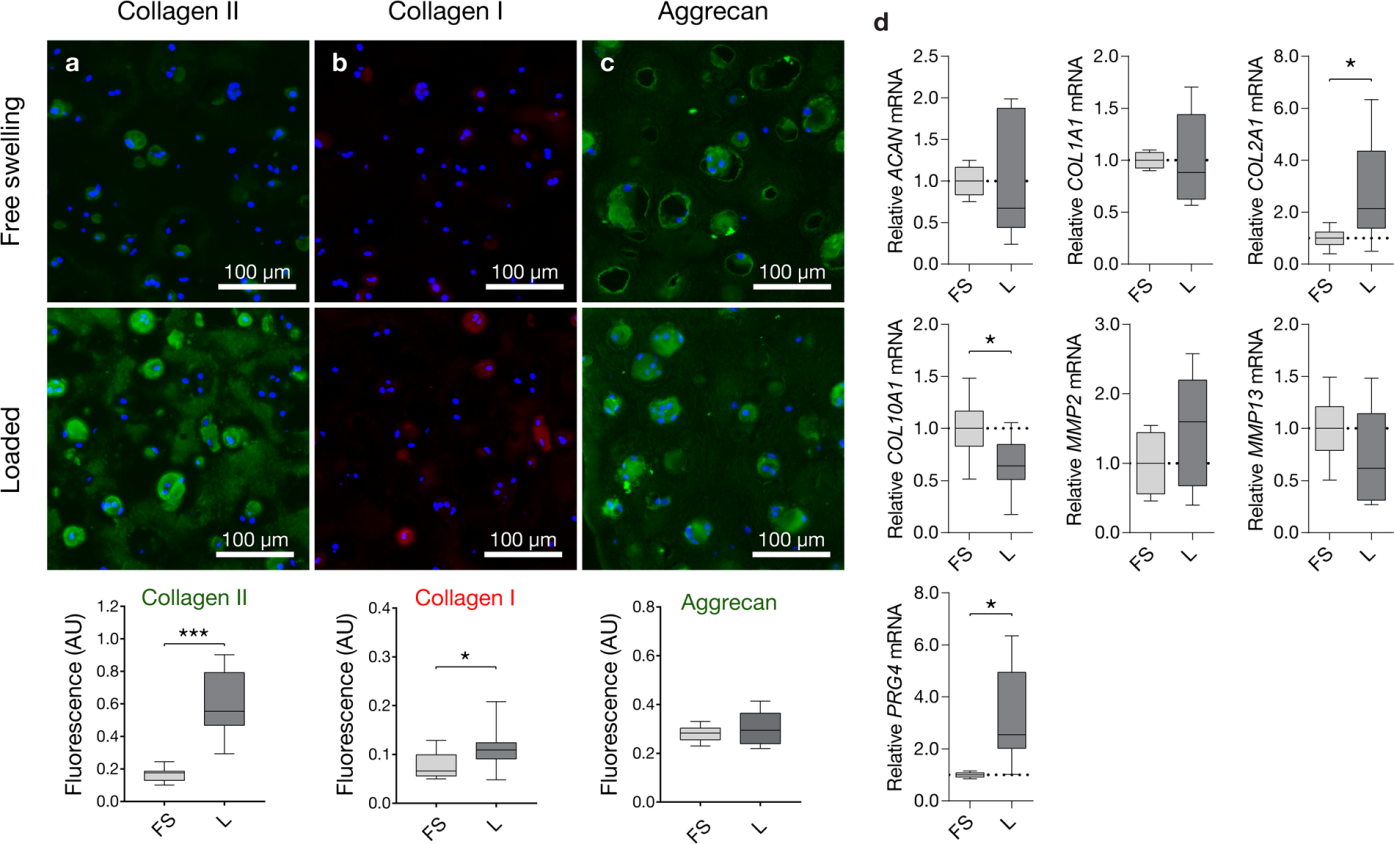
Extracellular matrix accumulation and chondrocyte gene expression in tissue-engineered cartilage constructs. Constructs were pre-cultured for 14 days in free swelling conditions, followed by 14 days of additional free swelling culture (FS) or intermittent biaxial mechanical stimulation at 30% compressive strain and 1 mm shear amplitude (L) (1 Hz, 1 h daily). Immunofluorescence staining and integrated fluorescence intensities for (**a**) collagen II, (**b**) collagen I, and (**c**) aggrecan of statically cultured (FS) and loaded constructs (L). Immunoreactive regions for collagen II and aggrecan appear green, while immunoreactive regions for collagen I appear red. Nuclei were counterstained with DAPI (blue). (**d**) Relative gene expression levels 2 h after the last loading cycle. Gene expression levels of loaded constructs (L) were normalized to free swelling controls (FS) cultured for the same duration (mean of FS controls indicated by broken line). Stars indicate a statistical difference between free swelling and loaded constructs (*p < 0.05, ***p < 0.001) (3 donors, 2 construct each).

**Figure 7:**
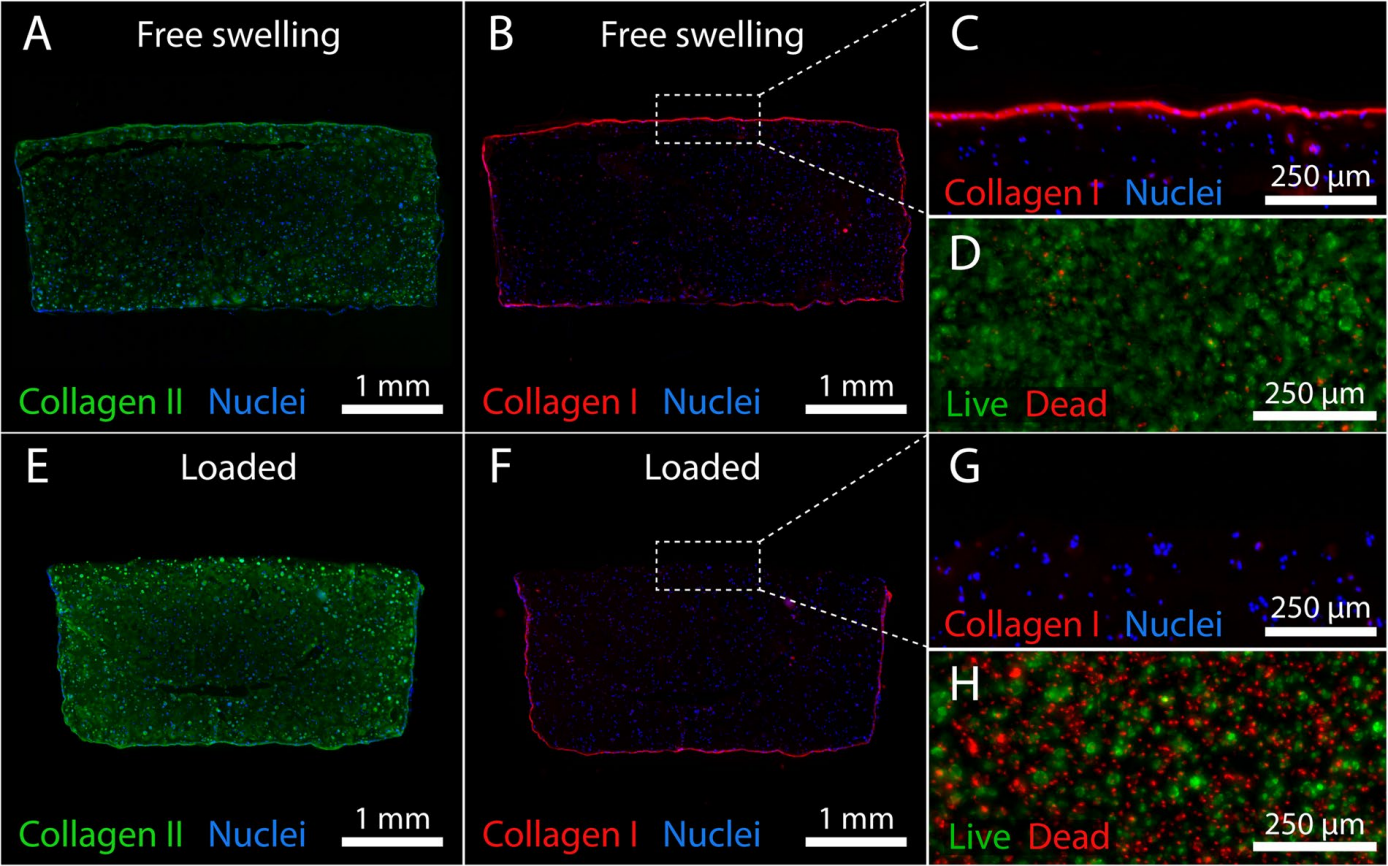
Effects of biaxial loading on collagen deposition in GelMA-HAMA constructs and viability of surface-adherent chondrocytes. Immunofluorescence staining for (**A**–**E**) collagen II and (**B**,**C**,**F**,**G**) collagen I in (**A**–**C**) statically cultured and (**E**–**G**) intermittently loaded constructs after 28 days. Immunoreactive regions for collagen II appear green, while immunoreactive regions for collagen I appear red. Nuclei were counterstained with DAPI (blue). Viability of chondrocytes adherent to the outer surface of a GelMA-HAMA construct at day 14 under (**D**) free swelling conditions or (**B**) after 1 hour of dynamic biaxial mechanical loading (1 Hz, 30% compressive strain, 1.5 mm shear amplitude). Living cells appear green, while dead cells appear red (viability stainings presented in panels (**D**) and (**H**) were performed in a separate, short-term experiment).

GelMA-HAMA constructs supported the synthesis and accumulation of large quantities of hyaline cartilage-like ECM. Immunoreactivity for collagen II was significantly higher (Fig. 6a) and appeared more homogenously distributed throughout the centre and periphery of mechanically stimulated constructs compared to free swelling controls (Fig. 7A,E). While reactivity for collagen I was minor in both culture conditions, marginally stronger intra- and pericellular staining was observed in loaded constructs (Fig. 6b). As previously reported^24,29^, strong collagen I immunoreactivity was observed at the outer surface of cell-hydrogel constructs (Fig. 7B,C), deposited by cells adherent to the gel surface displaying spreading and de-differentiated morphologies (Fig. 7D) similar to chondrocytes propagated on tissue culture plastic^30^. Interestingly, no immunoreactivity was observed on gel surfaces which were loaded (Fig. 7F,G). We hence investigated whether biaxial loading may cause death of surface-adherent cells using live-dead staining in a separate short-term experiment (Fig. 7D,H). Confirming this hypothesis, a dense monolayer of predominantly viable cells was observed on the outer surfaces of free swelling control construct (Fig. 7D), whereas loaded construct surfaces displayed high percentages of dead cells (Fig. 7H). Aggrecan immunostaining appeared strong in both free swelling and loaded tissue constructs, particularly in pericellular areas and more diffuse in interterritorial areas (Fig. 6c). Consistent with the GAG data presented in Fig. 5c,d, there was no significant effect of loading on the accumulation of aggrecan.

To investigate whether chondrocytes remain mechanosensitive after prolonged intermittent mechanical stimulation, we analysed chondrocyte gene expression in free swelling and loaded cartilage constructs 2 h after the last loading cycle had ceased (Fig. 6d). At this time point, loading had no significant effect on the transcription of the chondrogenic marker *ACAN*, the de-differentiation marker *COL1A1*, as well as matrix metalloproteinases *MMP2* and *MMP13* which were expressed at statistically similar levels in both free swelling and loaded constructs. The expression of the chondrogenic markers *COL2A1* and *PRG4*, however, was significantly higher in mechanically stimulated constructs compared to controls, suggesting that an extension of the dynamic culture duration in future experiments may be beneficial. Interestingly, the expression of the hypertrophy marker *COL10A1* was down-regulated following mechanical stimulation, indicating that loading may inhibit the partial ECM mineralization previously reported for GelMA-HAMA constructs^29^.

## Discussion

*Ex vivo* tissue engineering of articular cartilage is a rapidly evolving research area with the potential to significantly improve the clinical management of cartilage disorders. Yet, the high manufacturing costs and variable outcomes of tissue-engineered cartilage products partially caused by a lack of automated and well-controlled bioreactor systems is impeding the clinical translation of research outcomes. Here, we developed a user-friendly bioreactor system capable of facilitating uni- or biaxial mechanical stimulation to cartilage neotissues in an automated, well-controlled, and monitored fashion. We demonstrate that our system can be employed to study the effects of individual uni- or biaxial loading parameters on chondrocyte cellular behaviour, permitting the investigation of the regulatory role of each specific mechanical factor on cartilage neotissue formation. Our long-term experiments demonstrate that intermittent biaxial mechanical stimulation improves the biochemical properties of engineered cartilage constructs, yielding tissues with hyaline-like composition and progressively increasing mechanical functionality.

Previously reported bioreactors used for studies on chondrocyte biology and cartilage tissue engineering include systems applying compressive loading^31,32^, hydrostatic pressure^33^, shear loading^13,34^, and hybrid bioreactors incorporating multiple loading regimes^15,17,35–37^. Most hybrid bioreactors, however, exhibit a number of limitations such as minor sample size capacity^15,35,37^, use of non autoclavable materials^15^, unsealed reactor chambers unsuitable for aseptic long-term culture^17,35,36^, or inability to measure loads applied to samples^15,35,38^. Other design approaches did not incorporate separate culture wells^15,38^. This precludes, for example, the investigation of secreted factors such as cytokines and proteins from individual samples or the testing of different pharmaceutical interventions in one set-up. Furthermore, the reviewed systems are not able to control the atmospheric oxygen tension in order to mimic the hypoxic environment typical for articular cartilage^15,17,35,36,38^. We aimed to address these limitations in our novel design which combines precision of loading and process monitoring facilitated by well-characterised commercial hardware. The system is user-friendly and provides capabilities for aseptic long-term culture, optional atmospheric oxygen control, sufficient sample capacity for larger studies, a high level of automation, and experimental flexibility. Furthermore, accurate mechanical testing (Fig. 1e) can be performed throughout the culture period to investigate the functional maturation of cartilage constructs without compromising sterility (Fig. 1e,f). Yet, some of the bioreactor features may be considered as limitations. For example, it is required that the height of all samples is similar to ensure homogenous loading profiles. Additionally, all samples in a given plate must be loaded with the same loading protocol. An alternative design compensating these limitations would have to incorporate multiple linear actuators individually loading single samples. However, this design would likely require substantially more hands-on-time as sample height and loading protocols would need to be individually determined and tailored. Additionally, such a system would also involve higher investment costs and greater susceptibility to hardware and software failure.

To assess the suitability of our bioreactor system for functional tissue engineering applications, we employed chondrocyte-laden GelMA-HAMA hydrogels which were previously shown to support chondrogenesis and the accumulation of hyaline cartilage-like ECM in static cultures^24,29^. First, we aimed to optimize some of the key culture and loading parameters in a series of short-term experiments (Fig. 3). The selection of suitable loading parameters was challenging since the biological response of chondrocytes to mechanical stimulation may be influenced by many intrinsically interconnected factors including the employed cell source, passage number and culture system, type of loading, strain magnitudes, loading duration, loading frequency, as well as the time point of loading and the various combinations of these factors^39^. We hence based the parameters employed in our optimization experiments on previously published reports which outlined stimulatory effects of intermittent loading for short durations at physiological frequencies, independent of the culture system used. Lee and Bader^40^, for example, found that static and low-frequency dynamic stimulation inhibited chondrocyte biosynthesis, while physiological frequencies of 1 Hz had stimulatory effects – a finding which was later confirmed by multiple independent research groups utilising dynamic stimulation in various culture systems^41–44^. Another key parameter which appears to be well-preserved between different culture systems is the duration of applied loading cycles. It is now generally accepted that short intermittent loading (1–5 hours/day) enhances chondrogenesis^6,39^, while continuous dynamic stimulation is likely to have no significant^45^, or only adverse effects on chondrogenesis^46^ in most culture systems. Previous studies also outlined that unconfined mechanical loading results in more homogenous mechanical signals throughout the tissue-engineered construct when compared to confined loading scenarios, in which substantial pressure and deformation gradients exist close to the porous indenter^47^. On the other hand, one of the parameters that appears to be highly dependent on the intrinsic properties of the employed culture systems is the mechanical strain level applied to tissue-engineered cartilage^39^. We hence focused our short-term experiments primarily on the optimization of the less well-preserved parameters including the static preculture duration and the magnitude of uni- and biaxial strain levels, while keeping the loading frequency and cycle duration constant at 1 Hz and 1 h, respectively. Consistent with our previously published results using alginate gels^28^, we found that an extended static preculture duration of 14 days was necessary to stimulate the expression of chondrogenic marker genes *ACAN* and *COL2A1* in response to loading (Fig. 5b), while mechanical stimulation after only 7 days of preculture resulted in down-regulation of chondrogenic markers (Fig. 5a). The inhibitory effects of loading at early time points are likely related to the absence of a protective pericellular matrix (PCM) and lack of cell-PCM interactions facilitating mechanotransduction^28^ and may instead constitute a cellular stress response^48^. When uniaxial compression (Fig. 3b) or shear (Fig. 3d) was applied after 14 days of preculture, however, chondrogenic marker gene expression generally increased with strain magnitude and the highest expression levels were observed at 50% compressive strain and 1.5 mm shear amplitude, respectively. Considering knee cartilage is estimated to only undergo approximately 7–10% compression during walking^49,50^ and excessive mechanical stresses are associated with chondrocyte and cartilage pathogenesis^51^, the anabolic response of chondrocytes to such high strain levels may seem surprising. Further emphasizing this point, early studies employing bovine cartilage explants outlined stimulatory effects of compression levels lower than 5% strain^5^, suggesting that hydrogel-encapsulated chondrocytes may benefit from similarly low strain levels. Yet, our earlier studies employing human chondrocyte-laden alginate demonstrated no significant effects on chondrogenic marker gene expression at such low strain levels^6^. To appropriately interpret the differences in cell response between native and engineered cartilage, it has to be considered that hydrogels typically exhibit Young’s moduli which are 1–2 orders of magnitude lower than the native cartilage^52^. The transmission of mechanical stresses to hydrogel-embedded chondrocytes, particularly in the early stages of neotissue development, is hence substantially lower than in the native tissue, rendering higher deformations necessary to induce similar anabolic effects. Owing to ECM accumulation and increasing mechanical properties, engineered tissues at later stages of development may accordingly benefit from more moderate loading regimes. Yet, we found that, although the mechanical properties of tissue-engineered constructs increased significantly over time (Fig. 5b), the transcriptional levels of the key chondrogenic markers *COL2A1* and *PRG4* remained elevated in intermittently stimulated constructs after a total culture duration of 28 days (Fig. 6d), suggesting that chondrocytes have not desensitized to loading and that a continuation of the chosen loading regime may yield further beneficial effects in future experiments.

Similar to our previously published results using chondrocytes encapsulated in alginate gels^6^, we found that, in response to only one hour of loading, mechanoresponsive genes remained upregulated for at least 4 hours after loading had ceased (Fig. 3c). However, when the loading cycle duration was extended to 12 hours, chondrogenic marker genes were expressed at lower levels compared to shorter loading durations^6^. Together, these data confirm previous findings^3^ that short intermittent loading applied in regular intervals may prevent cellular desensitization to mechanical stimuli observed in continuous loading scenarios. Accordingly, it may be of further benefit to increase the frequency of loading beyond 1 loading cycle per day in future studies.

Contrary to the expression of chondrogenic markers, we found that transcript levels of the de-differentiation marker *COL1A1* were decreased in all investigated uni- (Fig. 3d) and biaxial loading conditions involving shear (Fig. 3e). We hypothesized that this observation may primarily stem from the necrotic death of surface-adherent chondrocytes which secrete high levels of collagen I under free swelling conditions^24,29^ (Fig. 7B,C) rather than cells embedded inside the constructs which generally express only minor levels of collagen I protein (Fig. 6b)^1,24^, and hence performed viability staining on these de-differentiated subpopulations. Confirming our hypothesis, we detected substantially higher percentages of dead cells (Fig. 7H) and a lack of collagen I immunoreactivity on the outer surfaces of loaded constructs (Fig. 7F,G). This can be attributed to the mechanical ablation of surface-adherent cells by sliding shear motion. Loading regimes involving shear may thus aid in eliminating the fibrous ECM layer observed on the outer surface of GelMA-HAMA neocartilage constructs^24,27^.

While there is limited evidence pointing to differences in the cellular responses to shear and compression in tissue-engineered constructs^53^, it is still unclear whether combining both loading types produces better outcomes in terms of tissue quality than each one alone. Although both dynamic uniaxial compression and shear induce cyclic matrix strain, the resulting forces and flows within the constructs differ greatly between the two loading types^54^. Dynamic compression causes periodic volumetric change, in turn increasing radial fluid flow, mass transport, and hydrostatic pressure. Dynamic shear, on the other hand, does not cause significant changes to the sample volume and the associated effects on mass transport and hydrostatic pressure remain absent. Interestingly, in our culture system, there were no substantial differences in chondrogenic marker gene transcription between samples exposed to uniaxial shear or compressive stimulation, nor to biaxial stimulation (Fig. 3). This is in line with another study^54^ and leads us to believe that the differential regulation of investigated marker genes is predominantly facilitated by chondrocyte mechanotransduction from cyclic matrix strain, rather than hydrostatic pressures or simply an improvement of nutrient and waste transport. Since we only investigated the transcriptional response of chondrocytes to uni- and biaxial loading in short-term experiments, additional long-term experiments are needed to more conclusively investigate whether uni- or biaxial loading yields better neotissue qualities in our dynamic culture system. However, this work was beyond the scope of the current study.

Although gene expression analysis indicated that short-term biaxial loading at 30% compressive strain and 1.5 mm shear amplitude had the greatest effect on chondrogenic marker gene transcription (Fig. 3e), first signs of wear-and-tear were observed on a number of samples following only 1 h of loading. Since this was not the case at 30% compression and 1 mm shear amplitude, and gene expression profiles were comparable to harsher loading regimes (Fig. 3e), we decided to use this regime for our long-term experiments. We found that long-term intermittent biaxial loading had no significant effects on chondrocyte viability (Fig. 1a–d) and the total DNA content of tissue engineering constructs (Fig. 1e), suggesting that mechanical conditioning had no measurable cytotoxic effects on hydrogel-encapsulated chondrocytes and did not influence their proliferative behaviour.

The compressive properties of articular cartilage are highly dependent on fluid-pressurization facilitated by fixed negative charges of tissue-associated GAGs. Contents of GAG and aggrecan, a highly glycosylated proteoglycan abundant in articular cartilage, are hence commonly used markers of chondrogenesis and neocartilage formation. Although various investigated loading regimes significantly increased the transcription of *ACAN* over unstimulated controls in our short-term experiments (Fig. 3b–e), accumulation of aggrecan (Fig. 6c) and GAGs (Fig. 5c–f) remained similar between long-term intermittently loaded and statically cultured neocartilage constructs. In line with these findings, the Young’s moduli of stimulated and unstimulated neotissues increased similarly (approximately 2-fold) throughout the culture duration and no substantial differences were observed between both culture conditions. This may be related to the lack of transcriptional response of *ACAN* to long-term intermittent stimulation suggesting that the mechanosensitivity of this particular gene decreased over the culture duration, while other chondrogenic markers did not (Fig. 6d). Generally, it appears that the effects of mechanical stimulation on GAG accumulation and the physical properties of tissue-engineered cartilage are highly dependent on the implemented loading regimes and culture systems, but also the species and age of the chondrocyte donors^55–57^.

The synthesis and accumulation of collagen II, the most abundant structural component of articular cartilage, was highly dependent on the culture condition. Immunoreactivity in free swelling cultures was high, similar to our previous studies^24,29^. Remarkably, collagen II accumulation was further enhanced and appeared more homogenous in the interterritorial regions following intermittent mechanical loading (Figs 6 and 7). Collagen I immunofluorescence intensities were low within both free swelling and loaded constructs, but mechanical stimulation led to significantly higher levels compared to static controls (*p* = 0.023). However, immunoreactivity in loaded constructs was mainly localized to intra-/pericellular areas, while the reactivity in the interterritorial spaces appeared very low for both loaded and free swelling constructs (Figs 6 and 7). Similar to our previous studies^24,29^, direct comparison of collagen II and collagen I immunoreactivity (Figs 6 and 7) suggests that GelMA-HAMA constructs supported chondrogenesis of clinically relevant, expanded human chondrocytes and deposition of hyaline cartilage-like matrix, independent of the donor.

## Conclusion

We developed an innovative and user-friendly bioreactor system capable of applying defined uni- and biaxial mechanical stimulation to engineered cartilage neotissues in an automated, well-controlled, and monitored fashion to improve the quality of biological implants and reduce the associated manufacturing costs. We demonstrate that the system can be used to study the effects of individual loading and culture parameters on chondrocyte mechanobiology. We further demonstrate that intermittent biaxial stimulation of tissue-engineered constructs based on clinically relevant cells and biomaterials enhances the biosynthesis and accumulation of hyaline cartilage-specific ECM, yielding neotissues with superior native-like biochemical properties.

## Materials and Methods

### Bioreactor design and manufacture

The shear and compression bioreactor system was designed using SolidWorks 2012 3D CAD software (Dassault Systemes, SA, France). Manufacture and assembly were performed at the Queensland University of Technology Design and Manufacturing Centre (QUT DMC). The micro-linear actuators (T-NA Micro Linear Actuators, Zaber Technologies, Vancouver, Canada) and 50 N miniature load cell (LSB200, Futek, Irvine, USA) were purchased commercially.

### Actuator control and data acquisition

A custom virtual instrument and graphical user interface was developed using LabView (National Instruments, Austin, USA) to permit actuator control and acquisition of load and displacement data. The actuators and the load cell are connected to a laptop via USB without the need of additional control units.

### Macromer synthesis

GelMA^23^ and HAMA^24,58^ were synthesized as described previously. Briefly, gelatin (porcine skin, Type A, gel strength 300; Sigma Aldrich, St. Louis, MO, USA) was dissolved in phosphate-buffered saline (PBS, pH 7.4; Invitrogen, Carlsbad, CA, USA) at 10% w/v and reacted with 0.6 g of methacrylic anhydride (MAAh) per gram of gelatin for 1 h at 50 °C under constant stirring. Hyaluronic acid (HA, molecular weight 0.86 MDa, Novozymes, Denmark) was dissolved in PBS at 1% w/v and reacted with a 5-fold molar excess of MAAh over HA hydroxyl groups for 24 h on ice at pH 8. Insoluble MAAh and low molecular weight by-products were removed by dialysis against ultrapure water (MilliQ, Merck Millipore) and the pH of the dialysed products was adjusted to 7.4, after which macromer solutions were lyophilised and stored at −20 °C until use.

### Articular chondrocyte isolation and expansion culture

Human articular cartilage specimens were obtained from patients undergoing total knee arthroplasty surgeries for osteoarthritis. Chondrocytes were isolated from macroscopically normal cartilage of the lateral femoral condyles, as described elsewhere^59^. For short-term gene expression studies, chondrocytes were isolated from a total of 4 donors (60, 72 and 74 year old females, 56 year old male), while cells from 3 donors were used for long-term experiments (66 and 67 year old males, 65 year old female). Following isolation, cells were expanded on tissue culture plastic in low-D-glucose chondrocyte basal medium (Dulbecco’s modified Eagle’s medium (DMEM) with 2 mM GlutaMAX™, 10 mM 4-(2-hydroxyethyl)-1-piperazineethanesulfonic acid (HEPES), 0.1 mM nonessential amino acids, 50 U/mL penicillin, 50 µg/mL streptomycin, 0.5 µg/mL amphotericin B (Fungizone®) (all Invitrogen, CA, USA), 0.4 mM L-proline and 0.1 mM L-ascorbic acid (both Sigma-Aldrich)) supplemented with 10% foetal bovine serum (FBS) (Hyclone, Logan, UT, USA). All cell cultures/hydrogel constructs were incubated at 37 °C in a humidified 5% CO_2_/95% air CO_2_ incubator with the medium replaced every 3–4 days.

Ethical approval was granted for this research from the Queensland University of Technology Human Research Ethics Committee (EC00171) and the Prince Charles Hospital Human Research Ethics Committee (EC00168) (both Brisbane, Australia), and written informed consent was obtained from the donors for use of their tissue samples. All experiments were performed in accordance with the National Health and Medical Research Council (NHMRC) guidelines.

### Chondrocyte encapsulation and culture

GelMA and HAMA were dissolved in PBS containing 0.05% w/v Irgacure 2959 (1-[4-(2-hydroxyethoxy)-phenyl]-2-hydroxy-2-methyl-1-propanone; BASF, Ludwigshafen, RLP, Germany) at 37 °C. Passage 1 human articular chondrocytes were suspended in the hydrogel precursor solutions at 7–10 million cells/mL and photocrosslinked by exposure to 365 nm light at an intensity of ~2.6 mW/cm^2^ in a CL-1000 crosslinker (UVP, Upland, CA, USA) for 15 min in a custom polytetrafluoroethylene (PTFE) casting mold which produces constructs with dimensions of 4 mm × 4 mm × 2 mm (L × W × H). For short term experiments (Fig. 3), cells were encapsulated in 14.5% w/v GelMA and 0.5% w/v HAMA, while 10% w/v GelMA and 0.5% w/v HAMA was employed for long-term experiments to allow better comparison of neo-tissue formation under dynamic loading conditions with our previously published results obtained under static conditions using one chondrocyte donor^24,29^. Cell-hydrogel constructs were cultured in serum-free high-D-glucose basal chondrocyte medium (see above for composition) with ITS-G (100 × dilution), 1.25 mg/mL bovine serum albumin (BSA), 0.1 μM dexamethasone (all Sigma-Aldrich) and 10 ng/mL transforming growth factor beta 3 (TGF-β3) (GroPep, Adelaide, SA, Australia).

### Uni- and biaxial mechanical stimulation of cell-hydrogel constructs

For short-term gene expression experiments, constructs were precultured under free swelling conditions for 7 or 14 days, followed by dynamic uni- or biaxial mechanical loading for 1 h at a frequency of 1 Hz with various loading regimes outlined in Fig. 3 (n = 4–6 technical replicates, one donor per study). Samples were terminated for total RNA isolation 0 h, 2 h, or 4 h after loading had ceased, as indicated. For long-term experiments, constructs were either cultured under free swelling conditions for 28 days, or precultured for 14 days, followed by daily biaxial dynamic loading for 1 h at 1 Hz with a compressive strain of 30% of construct height and shear amplitude of 1 mm for another 14 days (3 donors, 2 constructs per donor for DNA and GAG analysis; 3 donors, 1 construct per donor for viability and immunofluorescence analysis; 3 donors, 2 constructs per donor for gene expression analysis). For all loading experiments, hydrogel constructs were loosely confined beneath the PTFE loading pistons by laser-cut medical grade silicone rings (0.5-mm high, with a 5 mm × 5 mm hole in the centre) fitted into the bottom of each well. A static offset compressive strain of approximately 10% was applied to ensure contact to all constructs. The bioreactor system was maintained in a standard 5% CO_2_/95% air CO_2_ incubator at 37 °C for all experiments.

### Cell viability assay

Live and dead chondrocytes were visualized with fluorescein diacetate (FDA) and propidium iodide (PI, both Sigma), respectively. Constructs were washed in PBS at room temperature (RT), followed by incubation with 10 μg/mL FDA and 5 μg/mL PI in PBS for 3 minutes at RT, and then washed again in PBS. Z-stack fluorescence images were captured using a Carl Zeiss Axio microscope, and cell viability was quantified in at least 6 images from 3 biological replicates, respectively, using an ImageJ script (National Institutes of Health, USA). Cell viability is expressed as percentage of living to total cells.

### Biochemical analysis

To analyse the DNA and glycosaminoglycan (GAG) content of cell-laden GelMA-HAMA constructs, samples were weighed and enzymatically digested in a two-step procedure by overnight incubation in phosphate-buffered EDTA (pH 7.1) containing 1 mg/mL hyaluronidase (Sigma) at 37 °C, followed by addition of 0.5 mg/mL proteinase K (Invitrogen) and overnight incubation at 56 °C. DNA concentration in the digests was measured using the Quant-iT™ PicoGreen® dsDNA quantification assay (Invitrogen). GAG concentrations were measured using the dimethyl-methylene blue (DMMB) assay at pH 1.5^60^ and obtained values were corrected using values for cell-free gels to determine the amount of GAGs produced by cells. GAGs secreted to the culture media were measured at the last media change and expressed as GAGs secreted to the media within 24 h of culture, with and without 1 hour of mechanical loading.

### Mechanical testing

The Young’s modulus of constructs submerged in PBS at 37 °C was measured in unconfined compression tests using an Instron 5848 microtester equipped with a 5_N load cell (Instron, Melbourne, VIC, Australia). Constructs were compressed at 0.01 mm/s using a non-porous aluminium indenter and the Young’s modulus was determined as the slope of the stress-strain curve from 10–15% strain^23^. To assess the bioreactor’s capabilities for mechanical testing, individual 15% w/v GelMA samples (n = 7) submerged in PBS at room temperature were compressed at 0.01 mm/s and the Young’s moduli determined between 10–15% strain were compared to moduli obtained with an Instron 5848 microtester using the same testing parameters. Cell-free GelMA hydrogels (15% w/v GelMA photocrosslinked for 15 min with 0.05% w/v Irgacure 2959; n = 6) were compressed to 10%, 30%, or 50% strain at 1 Hz to record representative force-displacement curves using the newly designed bioreactor system.

### Gene expression analysis

Cell-laden hydrogel constructs were homogenized in TRIzol reagent (Invitrogen), and total RNA was isolated according to the manufacturer’s instructions. Complementary DNA (cDNA) was synthesized using the SuperScript™ III First Strand Synthesis System (Invitrogen) with DNase and RNase digestions performed before and after cDNA synthesis, respectively. Quantitative reverse transcription real-time polymerase chain (qRT-PCR) was performed using SybrGreen® Mastermix (Invitrogen) and either a 7900HT fast real-time PCR system or QuantStudio™ 7 Flex Real-Time PCR system (both Applied Biosystems). The cycle threshold (C_t_) value of each gene was normalized to the geometric mean of the housekeeping genes *B2M* and *TBP* using the comparative C_t_ method (2^−ΔCt^). The forward (F) and reverse (R) primer sequences (5′→3′) used for PCR were as follows: *ACAN*: F:GCCTGCGCTCCAATGACT, R: TAATGGAACACGATGCCTTTCA; *B2M*:F:ATGAGTATGCCTGCCGTGTGA, R:GGCATCTTCAAACCTCCATGATG; *COL1A1*: F:CAGCCGCTTCACCTACAGC, R:TTTTGTATTCAATCACTGTCTTGCC; *COL2A1*: F:GGCAATAGCAGGTTCACGTACA, R:CGATAACAGTCTTGCCCCACTT; *COL10A1*: F:ACCCAACACCAAGACACAGTTCT, R:TCTTACTGCTATACCTTTACTCTTTATGGTGTA; *MMP2*:F:CCGTCGCCCATCATCAA, R:AGATATTGCACTGCCAACTCT; *MMP13*: F:ACTTCACGATGGCATTGCTG, R:CATAATTTGGCCCAGGAGGA; *PRG4*: F:GAGTACCCAATCAAGGATTATCA, R:TCCATCTACTGGCTTACCATTGC; *TBP*: F:GAGCCAAGAGTGAAGAACAGTC,  R:CATCACAGCTCCCCACCATATT.

### Immunohistochemistry

Constructs were embedded in Optimal Cutting Temperature compound (OCT) (Sakura, Finetek, Tokyo, Japan), sectioned at a thickness of 10 μm, and air-dried. Sections were fixed with ice-cold acetone for 10 min, air-dried, rehydrated in 50 mM BaCl_2_/100 mM Tris HCl buffer (pH 7.3) for 1 h, and incubated in 100 mM Tris HCl buffer (pH 7.3) for 5 min. Sections stained for collagen I and II were incubated with 0.1% w/v hyaluronidase (Sigma-Aldrich) at 37 °C for 30 min for antigen retrieval. Primary antibodies for aggrecan (969D4D11, Invitrogen; 1:400 dilution in PBS with 2% donkey serum), collagen type I (I-8H5, MP Biomed, Solon, OH, USA; 1:300 dilution in PBS with 2% goat serum), and collagen type II (II-II6B3, Developmental Studies Hybridoma Bank (DSHB), Iowa City, IA, USA; 1:150 dilution in PBS with 2% goat serum) were applied overnight at 4 °C in a humidified chamber. Secondary antibodies were diluted 1:150 in PBS with 2% goat/donkey serum and 5 µg/mL 4′, 6-diamidino-2-phenylindole (DAPI) (Invitrogen) and applied for 1 h in the dark (AlexaFluor^®^ 488-labelled donkey anti-mouse, AlexaFluor^®^ 488-labelled goat anti-mouse, AlexaFluor^®^ 594-labelled goat anti-mouse; all Jackson ImmunoResearch, West Grove, PA, USA). A mouse IgG isotype control antibody (Jackson ImmunoResearch; 1:1000 dilution) and secondary antibody only were used as negative controls. Following two washing steps in PBS and drying, sections were mounted with ProLong Gold (Invitrogen) and imaged using a Zeiss Axio epifluorescence microscope (Zeiss Axio Imager 2). Integrated fluorescence density measurements were performed using ImageJ (National Institutes of Health, USA) on at least 6 immunofluorescence images from corresponding areas in the centre and periphery of hydrogel constructs. Intensity values for ECM proteins were normalized to intensities obtained for DAPI staining to correct for differences in cell numbers between images.

### Statistical analysis

Statistical analysis was performed using GraphPad Prism software (version 7; GraphPad, CA, USA) with a significance level of 0.05. One-way analysis of variance (ANOVA) with Tukey’s post-hoc tests were performed to investigate the effects of culture duration and culture condition on chondrocyte viability and gene expression, as well as total DNA content, wet weight, and Young’s moduli of cell-laden constructs. The gene expression data presented in Fig. 3 were log_2_-transformed before statistical analysis to correct for skew in the dataset. Unpaired Student’s t-tests were performed to investigate differences in GAG and extracellular matrix synthesis, as well as gene expression levels between free swelling and loaded constructs at day 28 of culture. Statistical differences are indicated in figures using symbols.

## Electronic supplementary material

Supplementary Information

Supplementary Video V1

## References

[1] Solheim, E. *et al*. Results at 10–14 years after microfracture treatment of articular cartilage defects in the knee. *Knee Surg. Sports Traumatol. Arthrosc*., 10.1007/s00167-014-3443-1 (2014). 10.1007/s00167-014-3443-1 25416965

[2] Buckwalter JA, Mankin HJ (1998). Articular cartilage: degeneration and osteoarthritis, repair, regeneration, and transplantation. Instr. Course Lect..

[3] Hansmann J, Groeber F, Kahlig A, Kleinhans C, Walles H (2013). Bioreactors in tissue engineering - principles, applications and commercial constraints. Biotechnol. J..

[4] Atala A, Kasper FK, Mikos AG (2012). Engineering complex tissues. Sci. Transl. Med..

[5] Sah RL (1989). Biosynthetic response of cartilage explants to dynamic compression. J. Orthop. Res..

[6] Jeon JE, Schrobback K, Hutmacher DW, Klein TJ (2012). Dynamic compression improves biosynthesis of human zonal chondrocytes from osteoarthritis patients. Osteoarthritis Cartilage.

[7] Demarteau O (2003). Dynamic compression of cartilage constructs engineered from expanded human articular chondrocytes. Biochem. Biophys. Res. Commun..

[8] Schulz RM, Bader A (2007). Cartilage tissue engineering and bioreactor systems for the cultivation and stimulation of chondrocytes. Eur. Biophys. J..

[9] Angele P (2003). Cyclic hydrostatic pressure enhances the chondrogenic phenotype of human mesenchymal progenitor cells differentiated *in vitro*. J. Orthop. Res..

[10] Elder BD, Athanasiou KA (2009). Hydrostatic pressure in articular cartilage tissue engineering: from chondrocytes to tissue regeneration. Tissue Eng. Part B Rev..

[11] Demarteau O, Jakob M, Schafer D, Heberer M, Martin I (2003). Development and validation of a bioreactor for physical stimulation of engineered cartilage. Biorheology.

[12] Tran SC, Cooley AJ, Elder SH (2011). Effect of a mechanical stimulation bioreactor on tissue engineered, scaffold-free cartilage. Biotechnol. Bioeng..

[13] Waldman SD, Spiteri CG, Grynpas MD, Pilliar RM, Kandel RA (2003). Long-term intermittent shear deformation improves the quality of cartilaginous tissue formed *in vitro*. J. Orthop. Res..

[14] Jin M, Frank EH, Quinn TM, Hunziker EB, Grodzinsky AJ (2001). Tissue shear deformation stimulates proteoglycan and protein biosynthesis in bovine cartilage explants. Arch. Biochem. Biophys..

[15] Yusoff N, Abu Osman NA, Pingguan-Murphy B (2011). Design and validation of a bi-axial loading bioreactor for mechanical stimulation of engineered cartilage. Med. Eng. Phys..

[16] Di Federico E, Bader DL, Shelton JC (2014). Design and validation of an *in vitro* loading system for the combined application of cyclic compression and shear to 3D chondrocytes-seeded agarose constructs. Med. Eng. Phys..

[17] Frank EH, Jin M, Loening AM, Levenston ME, Grodzinsky AJ (2000). A versatile shear and compression apparatus for mechanical stimulation of tissue culture explants. J. Biomech..

[18] Khademhosseini A, Langer R (2016). A decade of progress in tissue engineering. Nat. Protoc..

[19] Martin I, Wendt D, Heberer M (2004). The role of bioreactors in tissue engineering. Trends. Biotechnol..

[20] Athanasiou, K. A., Darling, E. M. & Hu, J. C. *Articular Cartilage Tissue Engineering*. 182 (Morgan & Claypool, 2009).

[21] Place ES, Evans ND, Stevens MM (2009). Complexity in biomaterials for tissue engineering. Nat. Mater..

[22] Nichol JW (2010). Cell-laden microengineered gelatin methacrylate hydrogels. Biomaterials.

[23] Loessner D (2016). Functionalization, preparation and use of cell-laden gelatin methacryloyl-based hydrogels as modular tissue culture platforms. Nat. Protoc..

[24] Levett PA (2014). A biomimetic extracellular matrix for cartilage tissue engineering centered on photocurable gelatin, hyaluronic acid and chondroitin sulfate. Acta Biomater..

[25] Ragan PM (1999). Down-regulation of chondrocyte aggrecan and type-II collagen gene expression correlates with increases in static compression magnitude and duration. J. Orthop. Res..

[26] Ragan PM (2000). Chondrocyte extracellular matrix synthesis and turnover are influenced by static compression in a new alginate disk culture system. Arch. Biochem. Biophys..

[27] Buschmann MD, Gluzband YA, Grodzinsky AJ, Hunziker EB (1995). Mechanical compression modulates matrix biosynthesis in chondrocyte/agarose culture. J. Cell. Sci..

[28] Jeon JE (2013). Effect of preculture and loading on expression of matrix molecules, matrix metalloproteinases, and cytokines by expanded osteoarthritic chondrocytes. Arthritis Rheum..

[29] Levett PA, Hutmacher DW, Malda J, Klein TJ (2014). Hyaluronic acid enhances the mechanical properties of tissue-engineered cartilage constructs. PLoS One.

[30] Darling EM, Athanasiou KA (2005). Rapid phenotypic changes in passaged articular chondrocyte subpopulations. J. Orthop. Res..

[31] Palmoski MJ, Brandt KD (1984). Effects of static and cyclic compressive loading on articular cartilage plugs *in vitro*. Arthritis Rheum..

[32] Klein TJ, Malda J, Sah RL, Hutmacher DW (2009). Tissue engineering of articular cartilage with biomimetic zones. Tissue Eng. Part B Rev..

[33] Elder BD, Athanasiou KA (2009). Effects of temporal hydrostatic pressure on tissue-engineered bovine articular cartilage constructs. Tissue Eng. Part A.

[34] Meinert C (2017). Tailoring hydrogel surface properties to modulate cellular response to shear loading. Acta Biomater..

[35] Grad S (2005). Surface motion upregulates superficial zone protein and hyaluronan production in chondrocyte-seeded three-dimensional scaffolds. Tissue Eng..

[36] Waldman, S. D., Couto, D. C., Grynpas, M. D., Pilliar, R. M. & Kandel, R. A. Multi-axial mechanical stimulation of tissue engineered cartilage: review. *Eur. Cell Mater*. **13**, 66–73; discussion 73–64 (2007). 10.22203/ecm.v013a07 17429796

[37] Shahin K, Doran PM (2012). Tissue engineering of cartilage using a mechanobioreactor exerting simultaneous mechanical shear and compression to simulate the rolling action of articular joints. Biotechnol. Bioeng..

[38] Shahin K, Doran PM (2012). Tissue engineering of cartilage using a mechanobioreactor exerting simultaneous mechanical shear and compression to simulate the rolling action of articular joints. Biotechnology and Bioengineering.

[39] Natenstedt J, Kok AC, Dankelman J, Tuijthof GJM (2015). What quantitative mechanical loading stimulates *in vitro* cultivation best?. Journal of Experimental Orthopaedics.

[40] Lee DA, Bader DL (1997). Compressive strains at physiological frequencies influence the metabolism of chondrocytes seeded in agarose. Journal of Orthopaedic Research.

[41] Okuda Y, Konishi R, Miyata S (2013). Effect of Cyclic Compressive Stimuli on Mechanical Anisotropy of Chondrocyte-Seeded Agarose Gel Culture. Transactions of the Japan Society of Mechanical Engineers Series C.

[42] Hilz FM (2014). Influence of extremely low frequency, low energy electromagnetic fields and combined mechanical stimulation on chondrocytes in 3-D constructs for cartilage tissue engineering. Bioelectromagnetics.

[43] Elder BD, Athanasiou KA (2008). Synergistic and additive effects of hydrostatic pressure and growth factors on tissue formation. PLoS One.

[44] Shelton, J. C., Bader, D. L. & Lee, D. A. Mechanical conditioning influences the metabolic response of cell-seeded constructs. *Cells Tissues Organs* **175**, 140–150, doi:74630 (2003). 10.1159/000074630 14663157

[45] Torzilli PA, Bhargava M, Chen CT (2011). Mechanical Loading of Articular Cartilage Reduces IL-1-Induced Enzyme Expression. Cartilage.

[46] Torzilli PA, Tehrany AM, Grigiene R, Young E (1996). Effects of misoprostol and prostaglandin E2 on proteoglycan biosynthesis and loss in unloaded and loaded articular cartilage explants. Prostaglandins.

[47] Hung CT, Mauck RL, Wang CC, Lima EG, Ateshian GA (2004). A paradigm for functional tissue engineering of articular cartilage via applied physiologic deformational loading. Ann. Biomed. Eng..

[48] Jean C, Gravelle P, Fournie JJ, Laurent G (2011). Influence of stress on extracellular matrix and integrin biology. Oncogene.

[49] Eckstein F (2005). *In vivo* cartilage deformation after different types of activity and its dependence on physical training status. Ann. Rheum. Dis..

[50] Chan DD (2016). *In vivo* articular cartilage deformation: noninvasive quantification of intratissue strain during joint contact in the human knee. Sci. Rep..

[51] Coleman MC, Ramakrishnan PS, Brouillette MJ, Martin JA (2016). Injurious Loading of Articular Cartilage Compromises Chondrocyte Respiratory Function. Arthritis Rheumatol.

[52] Shepherd DE, Seedhom BB (1999). The ‘instantaneous’ compressive modulus of human articular cartilage in joints of the lower limb. Rheumatology (Oxford).

[53] Schatti O (2011). A combination of shear and dynamic compression leads to mechanically induced chondrogenesis of human mesenchymal stem cells. Eur. Cell Mater..

[54] Fitzgerald JB, Jin M, Grodzinsky AJ (2006). Shear and compression differentially regulate clusters of functionally related temporal transcription patterns in cartilage tissue. J. Biol. Chem..

[55] Kock L, van Donkelaar CC, Ito K (2012). Tissue engineering of functional articular cartilage: the current status. Cell Tissue Res..

[56] Bian L (2010). Dynamic mechanical loading enhances functional properties of tissue-engineered cartilage using mature canine chondrocytes. Tissue Eng. Part A.

[57] Kock LM (2010). Tuning the differentiation of periosteum-derived cartilage using biochemical and mechanical stimulations. Osteoarthritis Cartilage.

[58] Smeds KA (2001). Photocrosslinkable polysaccharides for *in situ* hydrogel formation. J Biomed. Mater. Res..

[59] Klein TJ (2010). Long-term effects of hydrogel properties on human chondrocyte behavior. Soft Matter.

[60] Farndale RW, Buttle DJ, Barrett AJ (1986). Improved quantitation and discrimination of sulphated glycosaminoglycans by use of dimethylmethylene blue. Biochimica et Biophysica Acta (BBA) - General Subjects.

