# Supplementary material for: A novel bioreactor system for biaxial mechanical loading enhances the properties of tissue-engineered human cartilage
Source: Sci Rep. 2017 Dec 5;7:16997. doi: 10.1038/s41598-017-16523-x (PMC5717235; doi:10.1038/s41598-017-16523-x)
Supplement: Supplementary file 1 — Supplementary Information [file 41598_2017_16523_MOESM1_ESM.pdf]

A novel bioreactor system for biaxial mechanical loading enhances the properties of tissue-engineered human cartilage

Christoph Meinert[1,2], Karsten Schrobback[1], Dietmar W. Hutmacher[1,2], Travis J. Klein[1,2*]

[1] Institute of Health and Biomedical Innovation, Queensland University of Technology, Brisbane, Queensland, 4059, Australia

[2] Australian Research Council Industrial Transformation Training Centre in Additive Biomanufacturing, Queensland University of Technology, Brisbane, Queensland 4059, Australia

Supplementary Information

Supplementary Video V1. 3D animation illustrating the working principle of the novel shear and compression bioreactor system.
